# P-70. Outcome of Gram-Negative Periprosthetic Joint Infection after Primary and Revision Total Hip and Knee Arthroplasty

**DOI:** 10.1093/ofid/ofae631.277

**Published:** 2025-01-29

**Authors:** Pansachee Damronglerd, Omar M Abu Saleh, Nicholas Bedard, Douglas R Osmon

**Affiliations:** Faculty of Medicine Thammasat University, Rochester, Minnesota; Mayo Clinic Rochester, Rochester, Minnesota; Mayo Clinic, Rochester, Minnesota; Mayo Clinic, Rochester, Minnesota

## Abstract

**Background:**

Gram-negative bacteria (GNB) account for 5-23% of all periprosthetic joint infections (PJI), following total knee or hip arthroplasties (TKA, THA). Literature on the management of Gram-negative PJI is limited. This study aimed to describe our experience with the presentation, management, and outcomes of Gram-negative PJI.

**Table 1:**
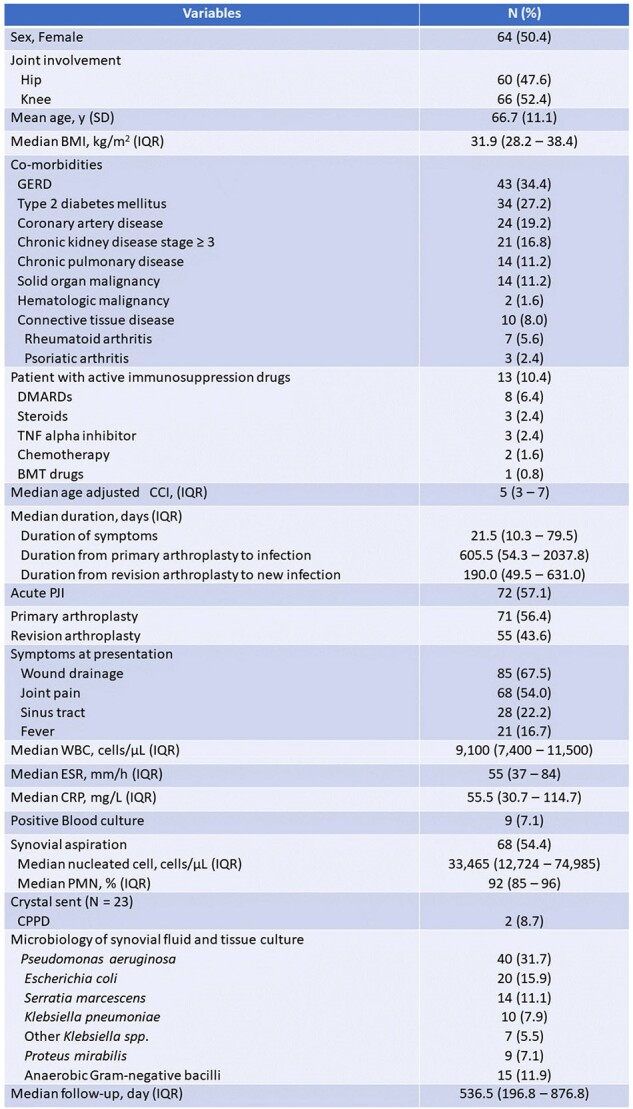
Baseline characteristics (N = 126 cases) Data are N (%) unless otherwise specified.

Abbreviations: BMT; Bone-marrow transplant, CCI; Charlson comorbidity index, CPPD; Calcium pyrophosphate crystal deposition, CRP; C-reactive protein, DMARDs; Disease-modifying antirheumatic drugs, ESR; Erythrocyte sedimentation rate, GERD; Gastroesophageal reflux disease, IQR; Inter-quartile range, PJI; prosthetic joint infection, SD; Standard deviation, TNF; Tumor necrotic factor, WBC; white blood cell

**Methods:**

A retrospective review of our infected institutional total joint registry from 2012-2023 identified 125 patients with 126 GNB PJI (60 THA and 66 TKA) according to MSIS criteria for PJI. Seventy-two cases (57%) of GNB PJI in this series were classified as acute based on the duration of symptoms of less than 4 weeks. Treatment failure was defined as reoperation for any reason, persistent infection, or reinfection post-surgery. Baseline demographics, presenting symptoms, microbiological data, and treatment outcomes were reviewed.

**Table 2:**
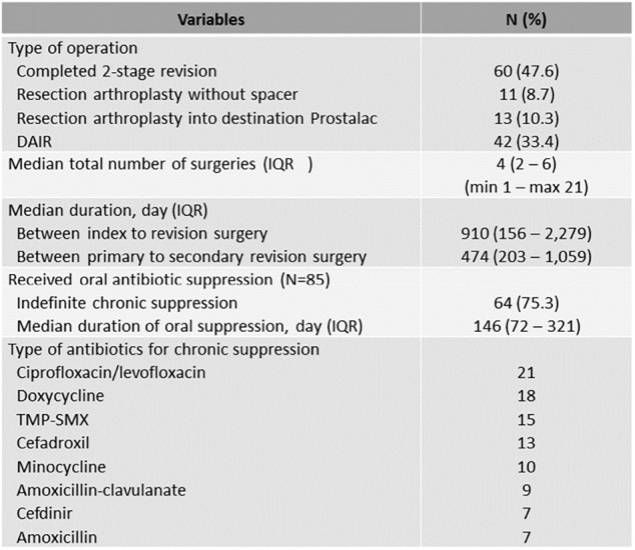
Therapies (N = 126 joints) Data are N (%) unless otherwise specified.

Abbreviations: DAIR; Debridement, antibiotics, and implant retention, IQR; Inter-quartile range

**Results:**

For baseline demographics, see Table 1. Notably, 10% of patients included in the study were immunosuppressed. Fifty-five cases (43.6%) had a history of revision prior to the diagnosis of PJI, including 44 cases with history of prior PJI. *Pseudomonas aeruginosa* was the most commonly isolated pathogen (31.7%). Wound drainage was associated with polymicrobial growth (40.8%). Debridement, antibiotics, and implant retention (DAIR) was utilized in 42 cases with 9.8% failure rate. History of revision arthroplasty, chronic PJI and the presence of sinus tract were associated with higher odds of reoperation within 1 year. Eighty-four cases underwent resection arthroplasty (RA), 73 had antimicrobial spacer, of those, 60 patients completed two-stage revision (TSR). At the time of reimplantation, 45/60 (75%) of cases had negative cultures (Table 2). The overall failure rate in resection group failure rate was 45.2%. Wound drainage was associated with higher odds of reoperation in this group (Table 3). The Failure rate in this cohort was 33%. Fluoroquinolones had a 90% susceptibility rate but were used infrequently for chronic suppression (25%).

**Table 3:**
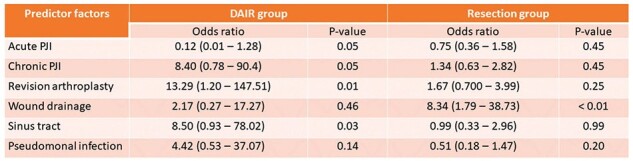
Predictor factors with reoperation for any reason within 1 year Data are N (%) unless otherwise specified. Abbreviations: DAIR; Debridement, antibiotics, and implant retention, PJI; Prosthetic joint infection

**Conclusion:**

*Pseudomonas aeruginosa* was the predominant pathogen in GNB PJI, with a high failure rate observed in the resection group. History of revision arthroplasty, chronic PJI, sinus tracts and wound drainage were associated with increased risk of reoperation within 1 year.

**Figure 1:**
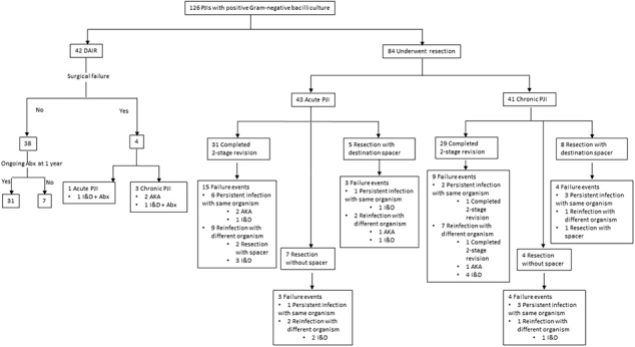
Distribution of failure events across the study

**Disclosures:**

**Nicholas Bedard, MD**, Stryker: Advisor/Consultant

